# Breaking the Cycle: A Yeast Mannan-Rich Fraction Beneficially Modulates Egg Quality and the Antimicrobial Resistome Associated with Layer Hen Caecal Microbiomes under Commercial Conditions

**DOI:** 10.3390/microorganisms12081562

**Published:** 2024-07-30

**Authors:** Aoife Corrigan, Paula McCooey, Jules Taylor-Pickard, Stephen Stockdale, Richard Murphy

**Affiliations:** 1 Alltech Bioscience Centre, A86 X006 Dunboyne, Co. Meath, Ireland; paula.mccooey@gmail.com (P.M.); rmurphy@alltech.com (R.M.); 2 Alltech (UK) Ltd., Stamford PE9 1TZ, UK; jpickard@alltech.com; 3 Novogene (UK) Company Ltd., 25 Cambridge Science Park, Cambridge CB4 0FW, UK; stephen.stockdale@novogene-europe.com; 4 BioFigR, Ballyvoloon, P24 N524 Cobh, Cork, Ireland

**Keywords:** poultry, layer, prebiotic, mannan-rich fraction, antibiotic resistance, metagenome, one health

## Abstract

Antibiotics and antibiotic growth promoters have been extensively employed in poultry farming to enhance growth performance, maintain bird health, improve nutrient uptake efficiency, and mitigate enteric diseases at both sub-therapeutic and therapeutic doses. However, the extensive use of antimicrobials in poultry farming has led to the emergence of antimicrobial resistance (AMR) in microbial reservoirs, representing a significant global public health concern. In response, non-antibiotic dietary interventions, such as yeast mannan-rich fraction (MRF), have emerged as a promising alternative to modulate the gut microbiota and combat the AMR crisis. This study investigated whether a yeast mannan-rich fraction containing feed supplement impacted the performance of laying hens, their microbiomes, and the associated carriage of antimicrobial resistance genes under commercial conditions. High-throughput DNA sequencing was utilised to profile the bacterial community and assess changes in the antibiotic resistance genomes detected in the metagenome, the “resistome”, in response to MRF supplementation. It was found that supplementation favourably influenced laying hen performance and microbial composition. Notably, there was a compositional shift in the MRF supplemented group associated with a lower relative abundance of pathobionts, e.g., *Escherichia*, *Brachyspira* and *Trueperella*, and their AMR-encoded genes, relative to beneficial microbes. Overall, the findings further demonstrate the ability of prebiotics to improve laying hen performance through changes associated with their microbiome and resistome.

## 1. Introduction

The gut microbiome can exert a profound influence on the health and physiology of its host [1]. In poultry, the gut microbiota plays a crucial role in fundamental functions such as nutrient digestion and absorption, energy metabolism, immune development, and protection against pathogens [2]. This significance has propelled the optimisation of the gut microbiome’s composition and functionality into a focal point for enhancing productivity and efficiency in laying hen production, a critical aspect of sustainable agriculture [3].

Over the years, antibiotics have been extensively employed in poultry farming to enhance growth performance, maintain bird health, improve nutrient uptake efficiency, and to prevent and control enteric diseases at both sub-therapeutic and therapeutic doses [4]. Unfortunately, this widespread use has led to the establishment of microbial reservoirs carrying antimicrobial resistance (AMR) determinants in poultry, posing a serious threat to global public health [5]. As a consequence, and in an effort to preserve antibiotic efficacy, numerous jurisdictions, including the European Union, New Zealand, and the USA, have banned the use of antibiotics as growth promoters, prompting poultry farmers worldwide to seek alternatives [6]. 

In response to the growing global threat of antibiotic resistance and the ban on antibiotic growth promoters, the quest for effective alternatives has gained urgency in food animal production. Several non-antibiotic dietary interventions that do not contribute selection pressures that promote the development of antibiotic resistance have emerged as promising strategies to modulate the gut microbiota and enhance host health and performance in the last decade [6]. Among these interventions, mannan-rich fractions (MRFs), derived from the cell wall of yeast, have attracted considerable attention due to their prebiotic properties, which favourably modulate the microbiome without conferring an antibiotic resistance-promoting selection pressure. 

MRFs, primarily composed of mannose and other polysaccharides, are known to exhibit prebiotic effects by serving as a substrate for beneficial bacteria, stimulating their growth and modulating the gut microbial community. For example, a meta-analysis showed benefits to the egg production, feed efficiency, and mortality of laying hens fed mannan-based prebiotics [7]. However, an important, yet comparatively unexplored, potential application of prebiotic MRF in layers is to improve bacterial diversity, impair pathobiont colonisation, and alter the bacterial resistome in the gastrointestinal tract. Recent research from a controlled environment study demonstrated favourable outcomes on bacterial diversity and uniformity, as well as pathogen control [8,9]. Other research has also shown that MRFs can potentiate bactericidal antibiotic efficiency, impacting antibiotic susceptibility in resistant strains of bacteria in vitro [10,11,12], with additional in vivo research highlighting reductions in resistant *Escherichia coli* and enterococci in broilers supplemented with MRF [13].

This study focused on investigating the impact of dietary supplementation with a yeast MRF-containing product on the caecal metagenome of laying hens under typical commercial conditions. Leveraging high-throughput DNA sequencing technologies, we comprehensively profiled the bacterial community in the caeca of laying hens fed the supplemented diet in comparison to control diets. Additionally, metagenomic analysis enabled the assessment of changes in the caecal resistome in response to MRF supplementation. Through the characterisation of alterations in caecal microbiota composition and antibiotic resistance gene profiles, our aim was to investigate whether MRF impacted the performance of laying hens, their microbiomes, and the associated carriage of antimicrobial resistance genes.

## 2. Materials and Methods

### 2.1. Ethics Statement

Animals were selected from commercial production units and were raised under animal welfare guidelines as set forth by the European Union. Birds were slaughtered in accordance with humane killing protocols as set forth in European Union Council Regulation (EC) 1099/2009.

### 2.2. Experimental Design, Sample Collection and Preservation

A layer trial was performed at a commercial production site in the United Kingdom. At 16 weeks old, 8000 pullets were randomly assigned to commercial free-range production units, where they received either a control standard commercial corn–soy mash diet or a standard diet plus a yeast mannan-rich fraction-containing supplement (Alltech Layer Pak™, Alltech Inc., Nicholasville, KY, USA) at the inclusion rate of 1 kg t^−1^ (Table S1). All other conditions were kept uniform among the groups. At 50 weeks old, the birds were housed permanently due to the threat of Avian Influenza. The birds were reared as per typical commercial production conditions, receiving feed and water ad libitum. Birds were culled at end of lay (77 weeks old), and the caecal contents of 12 randomly selected birds per shed (group) were aseptically transferred into sterile tubes containing 10 mL of DNAShield stabilisation solution (DNA/RNA Shield, Zymo Research, Irvine, CA, USA). The tubes were kept at room temperature for shipping and refrigerated thereafter. 

### 2.3. Performance Data

The average daily feed intake (ADFI), mortality (%), and percentage of second-grade eggs (%) was calculated for each 10-week period of the trial for weeks 20–29, 30–39, 40–49, 50–59, 60–69, and 70–76, as well as for the entire trial duration (weeks 20–76). The ADFI was calculated as the amount of feed delivered weekly, divided by the number of birds housed and the number of days per week. Mortality was calculated as percentage of birds dead per week. The percentage of graded seconds was calculated as the number of grade B eggs out of the total eggs produced weekly that were received by the egg packing centre. Eggshell strength was measured using an egg force reader (Orka food technology, West Bountiful, UT, USA), and eggshell strength (kgF) was measured as the average of 30 eggs per group per timepoint. The results were analysed using the unpaired Student’s *t*-test in GraphPad prism (*p* ≤ 0.05).

### 2.4. DNA Extraction, Library Construction, Quality Control, and Sequencing

DNA was extracted from caecal contents using a DNeasy Powersoil Pro kit (Qiagen, Hilden, Germany) according to the manufacturer’s instructions. The genomic DNA concentration, purity, and integrity were determined using an Agilent 5400 Fragment Analyzer System (Agilent Technologies, Santa-Clara, CA, USA). Sequencing libraries were generated using NEBNext^®^ Ultra™ DNA Library Prep Kit for Illumina sequencing (NEB, Ipswich, MA, USA). 

Whole DNA fractions were fragmented by sonication to the size of ~350 bp. The DNA fragments were then end-polished, A-tailed, and ligated to a full-length Illumina adaptor with PCR amplification. Each PCR product was purified (AMPure XP system), and library size distributions were established using an Agilent 2100 Bioanalyzer (Agilent Technologies, Santa-Clara, CA, USA)and quantified using a real-time PCR. A clustering of the uniquely indexed samples was performed on the Illumina cBot Cluster Generation System; then, the library preparations were sequenced on an Illumina NovaSeq 6000 platform, using an S4 flow cell with paired-end 150 bp reads (Novogene, Cambridge, UK).

### 2.5. Data Reprocessing and Assembly

The preprocessing of raw data from the Illumina sequencing platform was conducted with Readfq (V8, https://github.com/cjfields/readfq, accessed on 16 July 2024) to obtain clean data for subsequent analysis (Table S2). The specific steps are as follows: reads with low-quality bases were removed (default quality threshold < 38) above a certain proportion (default length is 40 bp); reads with N bases that reached a certain proportion (default length is 10 bp) and reads whose overlaps with adapters exceeded a certain threshold (default length is 15 bp) were removed. Considering the possibility of host contamination in the samples, the clean data were aligned to a reference host sequence, to filter out reads that may have come from a host origin using Bowtie2 software (version 2.2.4, http://bowtie-bio.sourceforge.net/bowtie2/index.shtml, accessed on 16 July 2024), with the following parameter settings: --end-to-end, --sensitive, -I 200, and -X 400 [14,15]. 

MEGAHIT software (v1.0.4-beta) was used for the assembly analysis of the clean data, with the assembly parameter settings --presets meta-large (--end-to-end, --sensitive, -I 200, -X 400), and Scaftigs without N were obtained by breaking the resulting Scaffolds from the N junction [16,17,18,19]. 

### 2.6. Gene Prediction and Abundance Analysis

With the default parameters, MetaGeneMark (V3.05, http://topaz.gatech.edu/GeneMark/, accessed on 16 July 2024) was used to perform an open reading frame prediction for Scaftigs (>= 500 bp) of each sample [14,15,20,21,22] using default parameters, and the information with a length less than 100 nt [16,17,18,23,24,25] in the prediction results was filtered out. For the ORF prediction results, CD-HIT [26,27] software (V4.5.8, http://www.bioinformatics.org/cd-hit/, accessed on 16 July 2024) was used to eliminate redundancy and obtain the non-redundant initial gene catalogue (with parameter settings [24]: -c 0.95, -G 0, -aS 0.9, -g 1, -d 0). Clean data of each sample was aligned to the initial gene catalogue by using Bowtie2 (Bowtie2.2.4) to calculate the number of reads of the genes on each sample alignment, with the following parameter settings [16,17,25]: --end-to-end, --sensitive, -I 200, -x 400. Genes with reads ≤2 [17,25] in each sample were filtered out to determine the gene catalogue (Unigenes) for subsequent analysis. Based on the number of reads aligned and the length of gene, the abundance of each gene in each sample was calculated by the following formula, in which *r* is the number of gene reads on alignment, and *L* is the length of gene [15,23,24,28,29,30]:

$$G_{k}=\frac{rk}{Lk}.\frac{1}{\Sigma_{i=1}^{n}\frac{ri}{Li}}$$

### 2.7. Species Annotation

Double index alignment of next-generation sequencing data [31] software (V0.9.9.110, https://github.com/bbuchfink/diamond/, accessed on 16 July 2024) was used for the alignment of Unigenes sequences with those of bacteria, fungi, archaea, and viruses extracted from the National Centre for Biotechnology Information (NCBI) non-redundant (NR) database (Version 2018-01-02, https://www.ncbi.nlm.nih.gov/, accessed on 16 July 2024), with the parameter settings blastp, e-value <= 1 × 10^−5^. From the alignment results of each sequence, the one with an e-value ≤ min. e-value × 10 is selected [15,22] was selected as the least common ancestor (LCA) algorithm applied to the Metagenome Analyzer (MEGAN) system classification of software to ensure the species annotation information of the sequence. According to the LCA annotation results and the gene abundance table, a table containing the number and abundance information of each sample in each classification level (phylum, class, order, family, genus, and species) was obtained.

On the basis of the abundance tables at each taxonomy level (Supplementary Tables S3–S8), a relative abundance overview and abundance clustering heatmap were performed, combined with a PCOA [32] (R ade4 package, Version 3.2.1), and NMDS using Bray–Curtis dissimilarity [33] (R vegan package, Version 2.15.3) analysis of dimension reduction (Table S9). Anosim analysis (R vegan package, Version 2.15.3) was used to test the differences between groups. Metastats and LEfSe analyses were used to search for species differences between groups. Metastats analysis was used to perform a permutation test between groups on each taxonomy level and obtain a *p*-value. Then, the Benjamini–Hochberg False Discovery Rate (fdr) was used to correct the *p*-value and obtain a q-value [34]. LEfSe software was used for the LEfSe analysis (LDA Score is 3 by default).

### 2.8. Resistance Gene Annotation

Resistance gene identifier (RGI) software was used to align Unigenes with the Comprehensive Antibiotic Resistance Database (CARD)4 [35,36,37] with the parameter set to Blastp (BLASTP), e-value ≤ 1 × 10^−30^. According to the comparison result, the relative abundance of the antibiotic resistance ontology (ARO) in all the genes was calculated as parts per million (ppm). According to the abundance of the ARO, the abundance bar graph and the number difference between the resistant genomes were displayed. In the same way, the abundance distribution of resistance genes in each sample, the species attribution analysis of resistance genes, and the resistance mechanism of resistance genes were analysed (BCD Table S10). The ARGs were assigned to the core resistome if the gene was present in all 16 samples. If a resistance gene was detected in at least one sample but less than 15 samples, it was assigned to the accessory resistome.

### 2.9. Bacterial Composition vs. Antimicrobial Resistance Genes

The genus relative abundance count table of shotgun sequencing-identified taxa and antibiotic resistance ontology files were processed identically using R, in the following specific order: Taxa or AMR genes needed to be (1) present in 12 of the 24 caecal samples, (2) present in the top 10% of the accumulative abundance of all taxa and genes identified, and (3) have a *p*-value difference < 0.1 between the control and supplemented group. The only difference in the preparation of the taxonomic and AMR data was the aggregation of duplicate AMR rows.

The corrplot package [v0.92] was used to calculate Spearman correlations between genera and AMR genes of interest using all observations. Taxa, followed by AMR genes, were hierarchically clustered, and this determined the order of the plotted variables. The correlation plot *p*-values shown were calculated within the corrplot package, whereas boxplot *p*-values were calculated using the ggpubr package [v0.6.0] “stat_compare_means” function, with the default Wilcoxon test selection. Boxplots were generated with ggplot2 [v3.4.4], with general data manipulation facilitated by the reshape2 package [v1.4.4].

## 3. Results

### 3.1. Performance

Results from the growth performance are shown in Table 1. Eggshell strength was significantly greater in the supplemented birds at weeks 30, 38, 45, 59, 63, and 75 and was 0.55 kgF greater overall from weeks 24 to 75 (*p* < 0.05). Mortality and ADFI were not significantly different between the control and supplemented birds but were lower over the entire feeding period by 19.05% and 2.3%, respectively, in the supplemented group. The rate of second-grade eggs was lower at weeks 30–39, 40–49, 50–59 and significantly greater at weeks 70–76 in the supplemented group compared to the control. Overall, the percentage of second-grade eggs was numerically lower by 7.07% over the entire laying period.

### 3.2. Sequencing and Microbial Diversity

**Figure 1:**
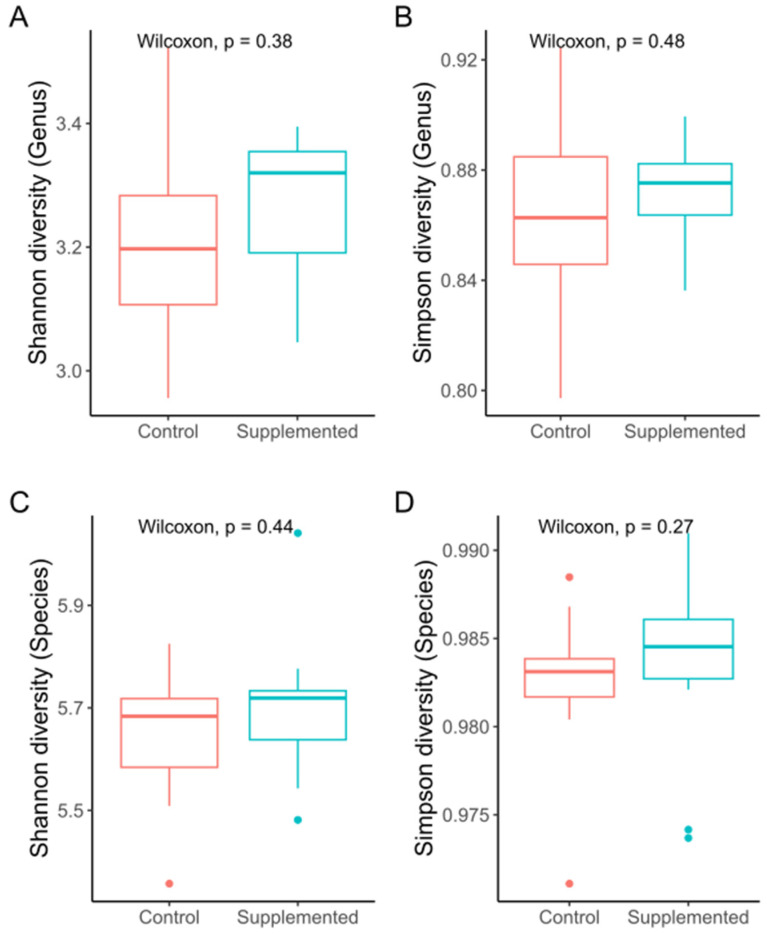
Impact of dietary supplementation on alpha diversity indices at Genus and Species level. Shannon H’ (**A**,**C**) and Simpson D (**B**,**D**) are shown for control and supplemented groups. Wilcoxon test group comparison *p*-values are shown.

DNA extracted from layer caecal content was sequenced with Illumina NovaSeq 6000, and after quality filtering, a total average number of 35,835,329 ± 1,334,350 high-quality sequences were analysed, resulting in a 99.97% percent clean-read rate (Table S2). After de novo metagenomic assembly by MEGAHIT, there were 71,757 to 315,613 assembled Scaftigs (Table S11). The Genus- (Figure 1A,B) and Species-level (Figure 1C,D) diversity estimates of the Shannon diversity and Simpson indexes were used to assess caecal microbial alpha diversity, and the results showed a trend towards greater diversity in the supplemented group when compared to the control, but this was not significantly different (Table S12). 

**Figure 2:**
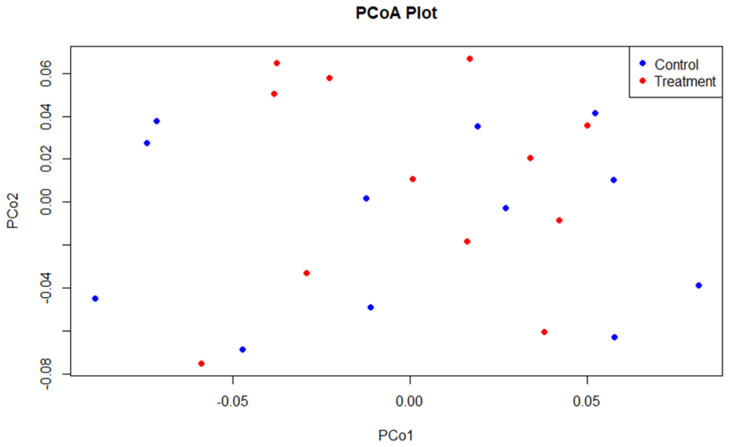
PCoA plot based on Bray–Curtis distance matrix at the genus level for control (blue) and supplemented (red) layers. Each point on the plot represents an individual sample.

The beta diversity, using principal coordinate analysis (PCoA), showed no significant differences (*p* = 0.485) in bacterial community structure between control and supplemented birds (Figure 2). 

### 3.3. Taxonomic Abundance and Composition of the Layer Caecal Microbiota

**Figure 3:**
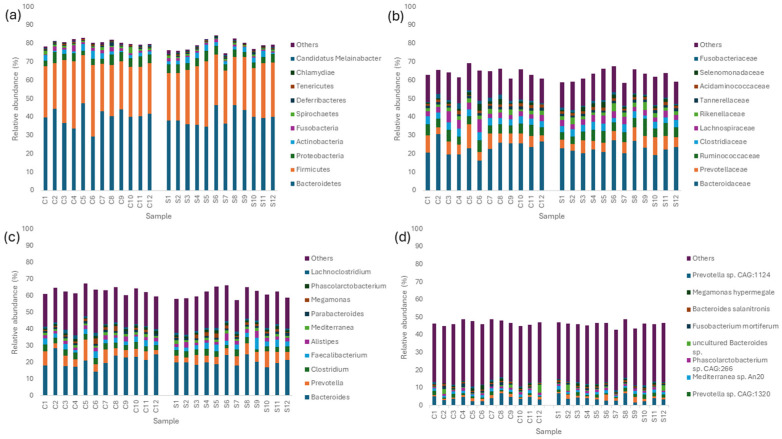
(**a**–**d**) Relative abundances of microbial communities in the control (J.C1-12) and supplemented (J.T 1-12) caecum of laying hens at phylum (**a**), family (**b**), genus (**c**), and species (**d**) levels. The *x*-axis represents the sample name. The *y*-axis represents the relative proportion of bacterial species annotated to a certain type. The legend on the right is the species category corresponding to each colour block. Diet is represented by C1–C12 for control group samples and S1–S12 for supplementedgroup samples.

**Figure 4:**
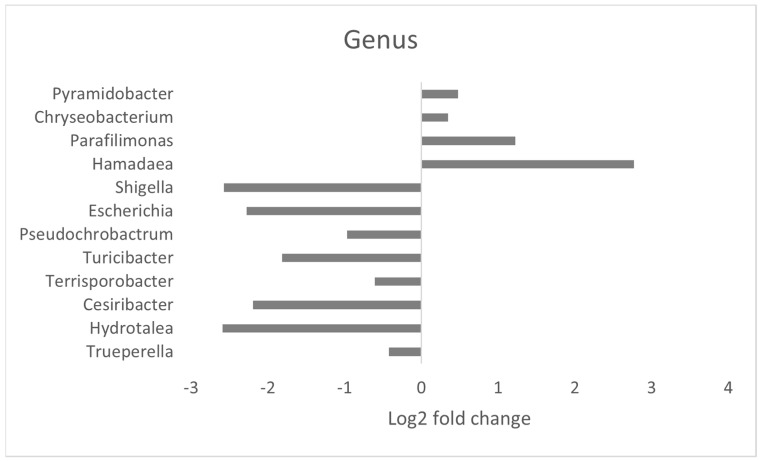
Caecal bacterial genera significantly different with dietary supplementation. Horizontal bars represent the log2-fold change of relative abundance, as determined by a differential abundance analysis performed using Metastats analysis, relative to the control (P_fdr_ < 0.005). Only genera with statistically significant differences in abundance are displayed.

**Figure 5:**
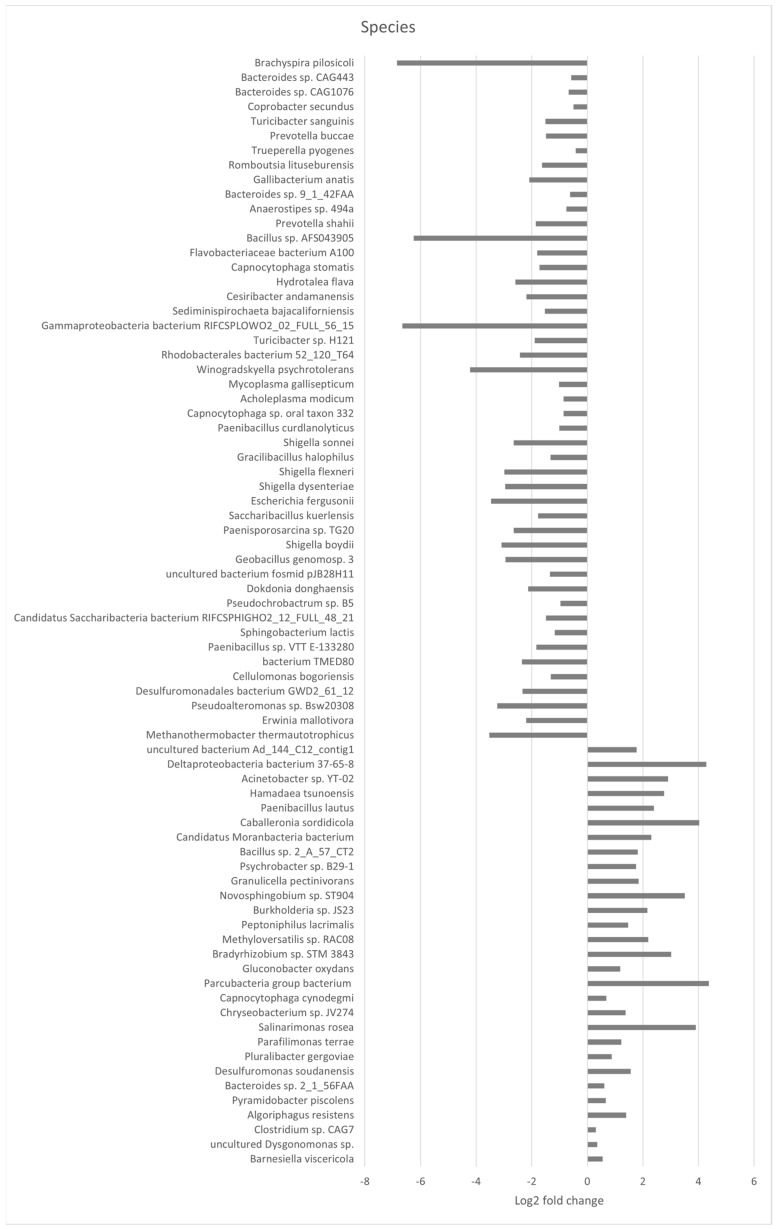
Caecal bacterial species significantly different with dietary supplementation. Horizontal bars represent the log2-fold change of relative abundance, as determined by a differential abundance analysis performed using Metastats analysis, relative to the control (P_fdr_ < 0.005). Only genera with statistically significant differences in abundance were displayed.

The abundance and composition of bacterial taxonomic groups at the phylum, family, genus, and species level are depicted in Figure 3a–d. The most prevalent phyla in the mature layer caecum were Bacteroidetes, Firmicutes, Proteobacteria, Actinobacteria, and Fusobacteria (Figure 3a). The most common families that were identified were Bacteroidaceae, Prevotellaceae, Ruminococcaceae, Clostridiaceae, and Lachnospiraceae (Figure 3b). Furthermore, the most abundant genera in the layer caecal content samples were *Bacteroides*, *Prevotella*, *Clostridium*, *Faecalibacterium*, and *Alistipes* (Figure 3c). Finally, the most prevalent species in layer caecal content were *Bacteroides* sp. An322, *Mediterranea* sp. An20, *Prevotella* sp. CAG:1320, *Phascolarctobacterium* sp. CAG:266, and *Alistipes* sp. CAG:831 in the control group and *Bacteroides* sp. An322, *Alistipes* sp. CAG:831, *Prevotella* sp. CAG:1320, *Mediterranea* sp. An20, and *Phascolarctobacterium* sp. CAG:266 in the supplemented group (Figure 3d). There were no significant differences observed between major bacterial phyla or families related to dietary supplementation. Bacterial genera which were differentially affected because of supplementation are shown in Figure 4 (Table S13). Thirteen bacterial genera were significantly different in abundance, with nine genera being significantly lower and four genera being significantly greater in the supplemented group. Seventy-six bacterial species were significantly different between the control and supplemented groups (Figure 5), with 29 species more abundant and 47 less abundant in the supplemented group (Table S14). 

**Figure 6:**
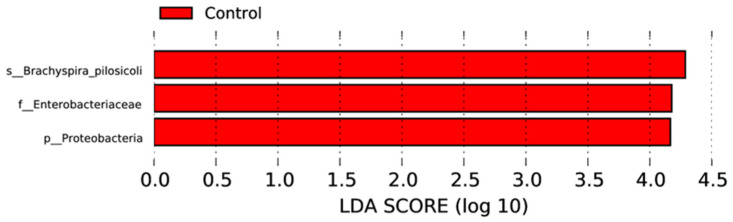
Distribution of linear discriminant analysis effect size results on the caecal microbiota of the control and supplemented laying hens. The LDA value distribution histogram shows the species whose LDA score is greater than the set value (the default setting is 4), that is, the biomarker with a significant difference between groups, and the length of the histogram represents the impact size of different species (i.e., LDA score).

Differentially abundant microbial communities were tested by linear discriminant analysis effect size, with a linear discriminant analysis score greater than 4.0. Representative bacteria at different taxonomic levels (from phylum to species) in the caecal microbiota of the supplemented and control groups are presented in Figure 6. The phylum proteobacteria, family Enterobacteriaceae, and species *Brachyspira pilosicoli* were more abundant in the control group caecal microbiota when compared with the supplemented group.

### 3.4. Effect of MRF Supplementation on Bacterial Pathobionts

**Figure 7:**
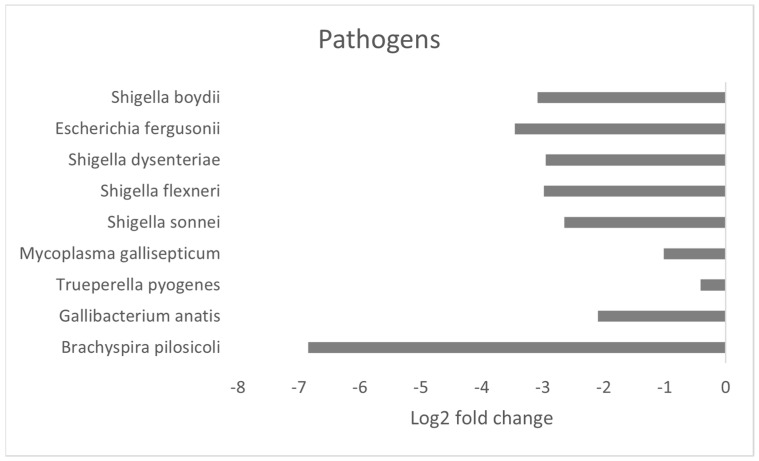
Caecal bacterial pathobionts significantly different with dietary MRF supplementation. Horizontal bars represent the log2-fold change of relative abundance as determined by a differential abundance analysis performed using Metastats analysis, relative to the control (P_fdr_ < 0.005). Only genera with statistically significant changes in abundance were displayed.

Among the bacterial genera and species that were differentially abundant between groups, we identified several pathobionts and examined the effect of dietary supplementation on their relative abundance. (Figure 7). At the genus level, the relative abundance of both *Escherichia* and *Shigella* were significantly lower in the supplemented group. When examining the bacterial species which were affected, nine were identified as potential pathobionts, including *Escherichia fergusonii*, *Shigella boydii*, *S. sonnei*, *S. Flexneri*, and *S. dysenteriae*. Additionally, *Brachyspira pilosicoli*, *Gallibacterium anatis*, *Mycoplasma gallisepticum*, and *Trueperella pyogenes* were all significantly lower in the supplemented groups. The total relative abundance of these bacterial pathobionts was substantially lowered with the MRF supplement relative to the control laying hens under commercial conditions (0.01% vs. 0.28%, respectively). 

### 3.5. Comparison of Antibiotic Resistance Genes between Control and MRF Supplemented Layers

**Figure 8:**
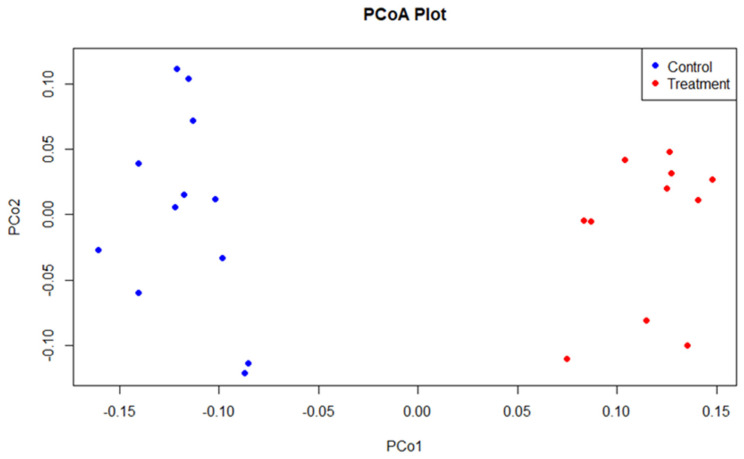
PCoA plot based on Bray–Curtis distance matrix of antibiotic resistance gene abundance for control (blue) and MRF-supplemented (red) layers. Each point on the plot represents an individual sample.

**Figure 9:**
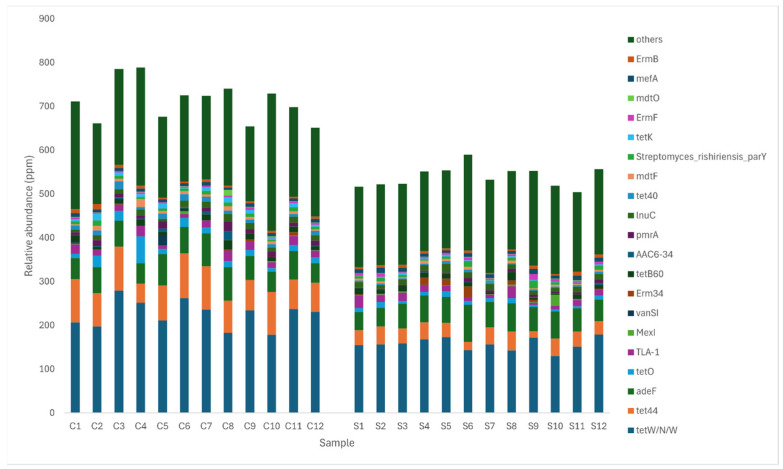
Histogram of the abundance of different AROs in each sample in control and MRF=supplemented layer caecal content. The relative abundance of all genes of ARO in each sample, in ppm, which is the result of enlarging the original relative abundance data by 106 times. Diet is represented by C1–C12 for control group samples and S1–S12 for supplemented group samples. Significantly fewer antibiotic resistance genes were detected in the caecal samples from the supplemented group (*p* < 0.001).

In total, 469 resistance genes were detected in layer caecal content. An analysis of beta diversity using PCoA based on Bray–Curtis distances and PERMANOVA between groups showed significant differences in ARG composition between the groups (Figure 8, *p* < 0.001). The 20 most abundant antibiotic resistance genes are shown in Figure 9. The resistance gene tetW/N/W was shown to be the most abundant across both control and supplemented caecal samples, followed by tet44, adeF, tetO, and TLA-1 in the control group and adeF, tet44, TLA-1, and lnuC in the supplemented group. The relative abundance of antibiotic resistance genes was significantly lower (*p* < 0.001) in the supplemented group (0.0539%) compared to the control group (0.0712%). According to the CARD database, all resistance genes identified in this study were classified into 10 primary categories based on their resistance mechanism: antibiotic efflux, antibiotic inactivation, antibiotic target alteration, antibiotic target protection, antibiotic target replacement, reduced permeability to antibiotics, and four multiple resistance mechanisms (antibiotic efflux and reduced permeability to antibiotics, antibiotic target alteration and antibiotic efflux, antibiotic target alteration and antibiotic efflux and reduced permeability to antibiotics, antibiotic target alteration and antibiotic target replacement). 

**Figure 10:**
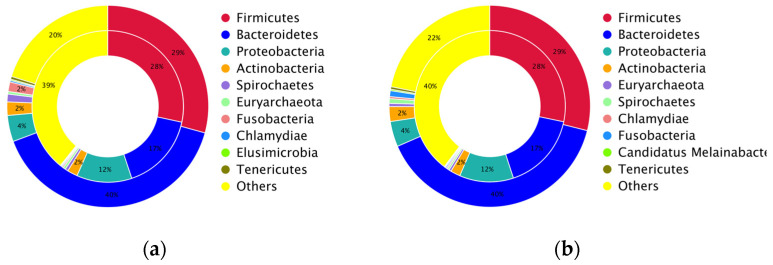
Circos plots representing alignment of the proportion of different antibiotic resistance ontologies and microbial phyla. Species identification analysis of AROs in the control (**a**) and MRF-supplemented (**b**) groups The inner ring refers to the distribution of different antibiotic resistance ontologies in corresponding microbial phyla. The outer ring refers to the relative abundance of different phyla in each group.

The dominant antibiotic resistance gene classes were found to be tetracyclines, fluoroquinolone multidrug, macrolide multidrug, aminoglycosides, and fluroquinolones in the control group and tetracyclines, fluoroquinolone multidrug, macrolide multidrug, aminoglycosides, and cephalosporin multidrug in the supplemented group. To compare the microbial origin of antibiotic resistance genes with the total microbial genes in the layer caecum, all antibiotic resistance genes and total gut microbial genes were classified into different taxa with the support of the resistance gene identifier. Different assignments of antibiotic resistance genes and total microbial genes at the phylum level were observed in both the control and supplemented group, 28% vs. 29% (Firmicuites), 17% vs. 40% (Bacteroidetes), and 12% vs. 4% (Proteobacteria), indicating that proteobacteria carried the greatest proportion of resistance genes compared with the other phyla (Figure 10). 

**Figure 11:**
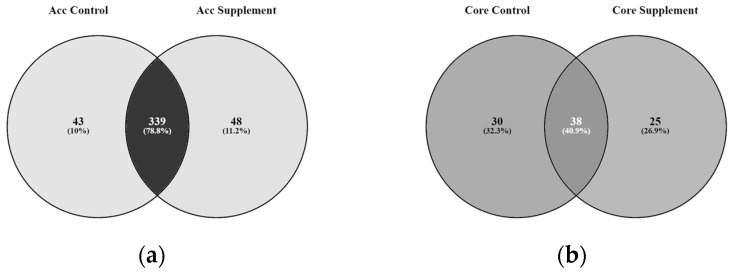
(**a**,**b**). Venn diagrams showing unique and shared genes of the core or accessory resistome for the control and supplemented groups.

From the total resistomes, ARGs were assigned to the core resistome if the gene was present in all 12 samples and to the accessory resistome if a gene was detected in at least 1 sample but less than 12 samples (Figure 11). A total of 68 ARGs were assigned to the core resistome and 382 ARGs were assigned to the accessory resistome for the control birds. In the supplemented birds, 63 ARGs were assigned to the core resistome and 387 ARGS were assigned to the accessory resistome. Within the core resistome, 30 genes were unique to the control and 25 genes were unique to the supplemented group, with 38 shared genes. Within the accessory resistome, 43 genes were unique to the control and 48 genes unique to the supplemented group, with 339 genes shared. 

A total of 49 ARGs were shown to be different between groups (P_fdr_ < 0.05, Table 2), with 41 genes being significantly lower in the supplemented group. Those genes that were significantly different between the groups were mainly associated with the following antibiotic resistance mechanisms: antibiotic efflux (25 genes); antibiotic inactivation (12 genes); antibiotic target alteration (5 genes); antibiotic target protection (4 genes); antibiotic target alteration and antibiotic efflux (2 gene); and one gene associated with antibiotic target alteration, antibiotic efflux and reduced permeability to antibiotics. These genes were associated with resistance to the following antibiotic classes: aminocoumarin antibiotic (2 genes), aminoglycoside antibiotic (3 genes), fluoroquinolone antibiotic (4 genes), Fosfomycin (2 genes), macrolide antibiotic (1 gene), penam antibiotic (1 gene), peptide antibiotic (3 genes), phenicol antibiotic (1 gene), streptogramin antibiotic (1 gene), tetracycline antibiotic (9 genes), or were multidrug resistant (22). The phylogenetic origins of the resistance genes which were significantly different between groups were assigned, and this indicated that many of those genes which were different between control and supplemented layers belonged to the phylum Proteobacteria, Enterobacteriaceae, and *Bacteroides* (Table 2). 

To further investigate the changes in microbial composition and AMR-encoded genes following MRF supplementation, the most abundant taxa detected across the study and differing between the control and supplemented group were analysed. A total of 29 genera were identified; however, one genus, Podovirus phage Cba41, was removed as it was deemed irrelevant to the subsequent AMR analyses performed. A total of 86 abundant AMR genes were found to substantially differ between the control and supplemented groups. For legibility, eleven lengthy CARD database AMR hits were simplified to represent the relevant gene name only.

**Figure 12:**
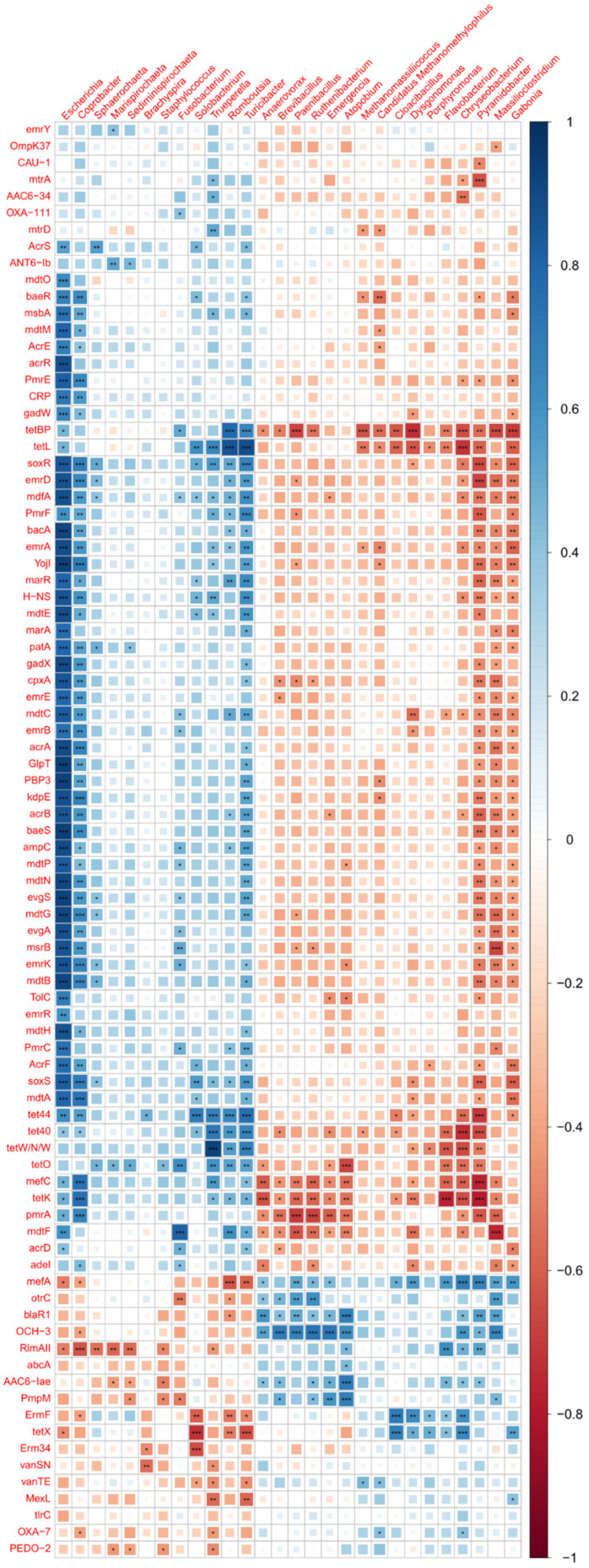
Spearman correlation analysis of abundant and significantly different taxa between the control and supplemented groups versus the abundant and significantly different antimicrobial resistance genes detected. The colour scale represents Spearman rho value rank correlations, with blue representing the strongest positive correlations. *p*-value legend: “***” ≤ 0.001, “**” ≤ 0.01, “*” ≤ 0.05.

**Figure 13:**
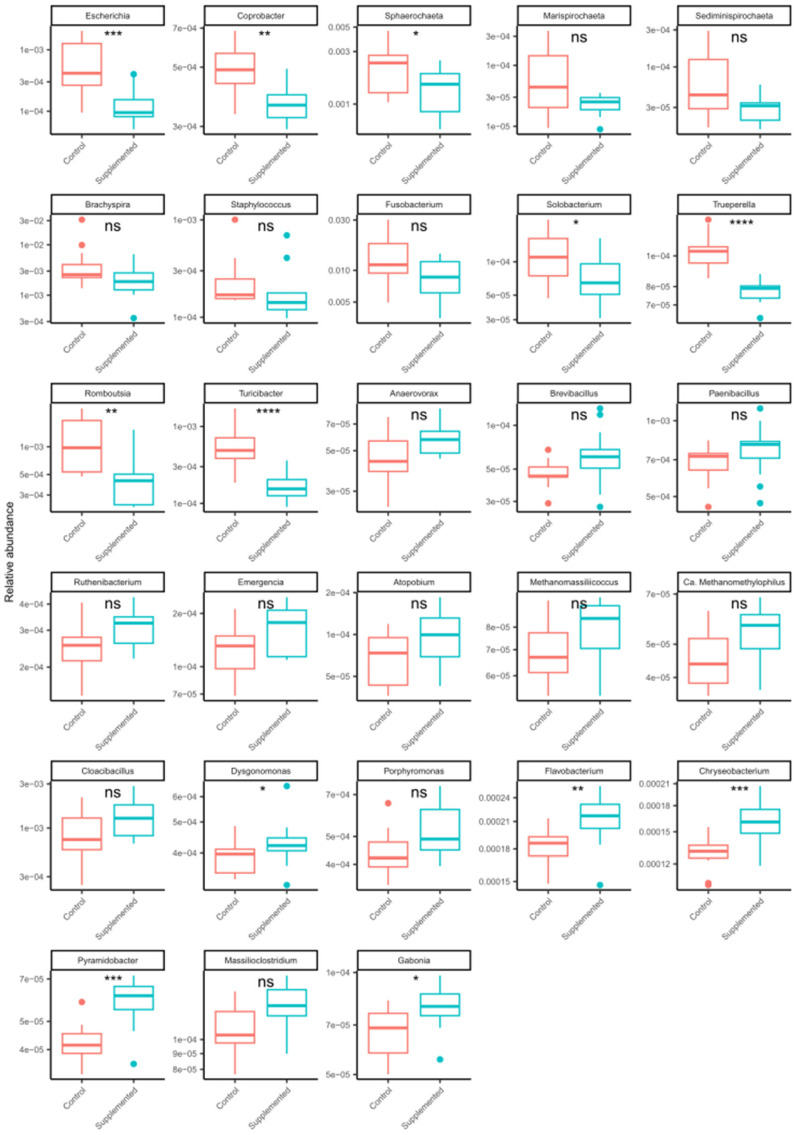
Individual taxon breakdown highlighting specific changes in relative abundance between the control and supplemented groups. Abbreviation “Ca.” represents “Candidatus”. The order of the boxplots corresponds to the order of taxa presented in Figure 12. *p*-value legend: “****” ≤ 0.0001, “***” ≤ 0.001, “**” ≤ 0.01, “*” ≤ 0.05, “ns” not significant.

The correlation analysis of the 28 bacterial genera versus the 86 AMR genes highlights strong and positive correlations between potential pathobionts and AMR genes, including *Escherichia*, *Coprobacter*, *Brachyspira*, and *Trueperella* (Figure 12). The multidrug efflux pump, YojI, was the strongest and most significant hit observed, associated with *Escherichia* (*p*-value 1.5 × 10^−11^). The fourth most significant hit was a tetracycline resistance mechanism, TetW/N/W, associated with pathobiont *Trueperella* (*p*-value 8.8 × 10^−11^). A subsequent focused analysis of the 28 most abundant genera showed MRF supplementation was significantly associated with decreases in *Escherichia*, *Coprobacter*, and *Trueperella* (Figure 13), whereas the decrease observed in the relative abundance of *Brachyspira* did not reach statistical significance.

**Figure 14:**
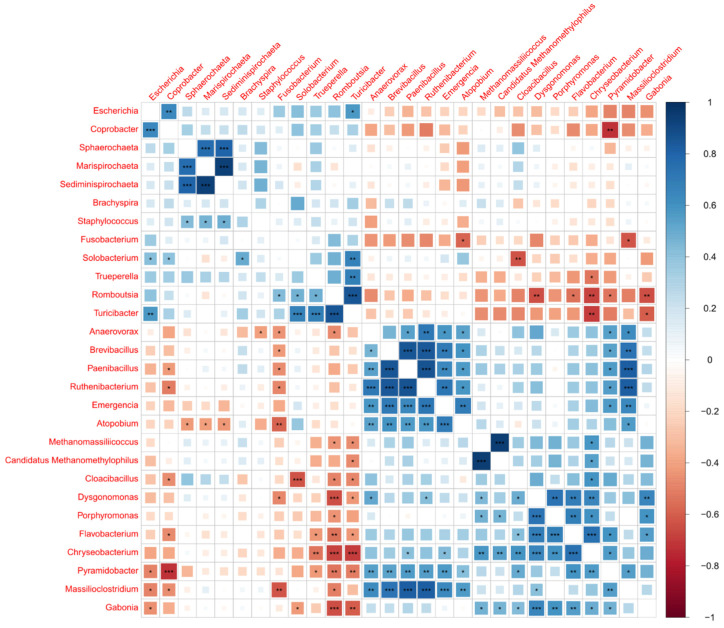
Spearman correlation analysis of abundant and significantly different taxa between control and supplemented groups versus themselves. The colour scale represents Spearman rho value rank correlations, with blue representing the strongest positive correlations. *p*-value legend: “***” ≤ 0.001, “**” ≤ 0.01, “*” ≤ 0.05.

Finally, a correlation analysis of the 28 most abundant and differing taxa between the control and supplemented groups highlights an increased abundance in anaerobic fermentative gut microbes that may also contribute to the inhibition of pathogens. *Anaerovorax*, *Brevibacillus*, *Paenibacillus*, *Ruthenibacterium*, *Emergencia*, *Atopobium*, strongly correlate together (Figure 14), with a consistent trend towards greater relative abundance in the MRF-supplemented laying hens. An expansion of these beneficial microbes could also be responsible for the observed decrease in relative abundance of AMR-carrying pathobionts.

## 4. Discussion

Nutritional strategies aimed at improving production performance and feed efficiency are pivotal for enhancing the economic viability of egg production. Dietary MRF has been extensively investigated in broiler chickens, and to a lesser extent, in laying hens, in relation to these production parameters. A published meta-analysis demonstrated that supplementing mannan-oligosaccharides in broiler diets led to notable improvements in body weight (+1.61%), feed conversion ratio (−1.99%), and mortality (−21.4%) compared to control diets [38]. Similarly, a recent meta-analysis of layers by [7] highlighted the positive impact of MRF supplementation on feed efficiency, mortality, egg mass, and eggshell thickness. Likewise, another recent study by Leigh and colleagues also demonstrated improved productivity factors in MRF-supplemented layers, such as total egg weight, total egg numbers, total egg mass, and laying frequency [9]. 

Consistent with the current literature, our commercial field-trial study showed a trend for lower mortality (−2.3%) and an improved ADFI (+19.05%) within the MRF-supplemented group, compared with the control group. The percentage of second-grade eggs was significantly lower in the supplemented groups during the majority of time periods (weeks 30–59, but not weeks 70–76). Combined over the entire laying period, the percentage of second-grade eggs was lower by 7.07% with MRF. In addition to this, eggshell strength (+12.09%) was significantly greater in the supplemented birds over the entire feeding period. 

Improvements in egg quality traits are important for enhancing the economic performance of laying hens, particularly in reducing egg cracking. Dietary nutrients such as calcium, phosphorous, trace minerals, and vitamins are important for the development of stronger shells. The observed positive effects on performance responses could be potentially attributed to better nutrient digestion and absorption facilitated by the improved intestinal development often associated with MRF supplementation, or a decrease in the need for the partition of nutrients toward supporting immune responses, thereby sparing more nutrients for production purposes [39]. 

Alpha and beta diversity indices often serve as indicators of gut health. In many scenarios, an increase in alpha diversity metrics is considered beneficial. Likewise, a beta diversity composition shift towards a microbiome composed of beneficial bacteria with fewer pathobionts is desired. Prebiotics such as yeast MRF have been found to have beneficial effects in broilers and layers in terms of improving bacterial diversity, decreasing the pathobiont load, and modulating immunity [8,9,40,41,42,43]. 

However, the results from this study showed that supplementation from 16 to 76 weeks of age did not significantly change alpha or beta diversity at the end of lay, which may be attributed to age-related variations in gut microbiota composition and stability. Factors influencing gut microbiota diversity include host and environmental factors, such as age and diet, and MRF supplementation at earlier stages of lay has been shown to effect both alpha and beta diversity indices [8,9]. Age-linked variations in the microbial composition of the layer intestinal tract have been defined in recent studies [44]. Longitudinal studies on the caecal microbiota of laying hens by [45,46] showed that bacterial diversity increased as birds matured. The composition of the gut microbiota fluctuates substantially before the laying period, while it is relatively stable in the laying period, which may be attributed to the management system of laying hens making the microbiome less susceptible to changes from dietary interventions as the hens age [44,47]. In the study by Wei et al. [45], dietary supplementation at later stages had no impact on diversity, as peak bacterial diversity was already achieved, suggesting the starter and mid-lay period are the key periods for the development of the gut microbiota in laying hens.

The importance of the gut microbiota regarding poultry growth performance and the reduction of pathogen load is well recognised. Similar to other studies of late-phase laying hens [47,48,49,50], we found that the caecal microbiota of our commercial laying hens was formed mainly by the phyla Bacteroidetes, Firmicutes, and Proteobacteria and genera *Bacteroides*, *Faecalibacterium*, and *Alistipes*. We further noted that there were significantly lower levels of numerous bacterial species in the MRF-supplemented group that are known to be disease causing, in both poultry and humans, specifically *Escherichia* and *Shigella*. The presence of normal flora in the intestine mainly functions to control or eliminate an invading pathogen, with the use of an in-feed prebiotic supplement strengthening the gut microbiota for improved host performance and colonisation resistance to pathogens.

A reduction in the levels of foodborne pathogens has also been noted in previous studies which involved the use of MRF in earlier stages of the life cycle of both laying hens and broilers [8,51]. MRF is known to bind type-1 fimbriated bacteria [52], and some of the pathogens that were affected in this study such as *Escherichia* spp., *Shigella* spp., and *Truperella pyogenes* are known to possess these mannose-sensitive adherence structures, which may explain their observed lower abundances in the supplemented group compared with the control [53,54,55]. Egg contamination with pathogenic bacteria can pose serious threats to food safety; therefore, modulating the intestinal microbiota is an effective way to reduce the risk of spreading foodborne pathogens in humans.

*Brachyspira pilosicoli*, the causative agent of avian intestinal spirochaetosis (AIS), was also lower in the supplemented group. Symptoms of infection can vary but typically present as reduced growth rates, delayed onset of lay, reduced egg production, faecally stained eggs, and diarrhoea. AIS has an estimated cost to the UK laying industry of GBP 18 million per annum; therefore, reducing the levels on-farm could have potential economic benefits for producers [56]. In addition, antimicrobials are the main control strategy for AIS in poultry, so alternative nutritional approaches to infection control could aid in reducing the use of antimicrobials in poultry [44,57]. 

While the emphasis of nutritional supplementation typically centres on control of bacteria within the intestinal tract causing gastrointestinal disease or presenting food safety implications, we observed in this study marked variations in some bacteria associated with respiratory pathologies. The avian respiratory tract is a common site of pathogen entry and disease, including both viruses and bacteria. The treatment and prevention of respiratory infections are of utmost importance for the industry, not only because they have a devastating effect on poultry flocks, but they also render flocks immunosuppressed and susceptible to opportunistic infections such as colibacillosis [58,59,60].

Significantly lower levels were noted for *Gallibacterium anatis* and *Mycoplasma gallisepticum* in the supplemented layer caeca, and given the potential for faecal–oral transmission of these diseases, this could potentially lower the risk of transmission and disease. Disease caused by *G. anatis* can lead to economic losses in the poultry industry due to respiratory issues in birds, as well as a range of pathological lesions in the ovaries and oviducts of laying hens, where it is estimated that infection of the reproductive tract can lower egg production by 8–10% [61]. *Mycoplasma gallisepticum* is a pathogenic mycoplasma species in poultry and can result in breathing difficulty, nasal discharge, sinusitis, airsacculitis, a decrease in egg production, and an increase in embryo mortality in layer parents and commercial layers [62]. Although infections with this pathogen may be subclinical, they can predispose birds to secondary infections with bacteria such as *Escherichia coli*, and viruses such as Newcastle disease, infectious bronchitis, and infectious laryngotracheitis. 

Antimicrobial resistance genes are ubiquitous in both the GIT and other environmental microbial reservoirs, and infections caused by antibiotic-resistant bacteria are a major threat to public health [63,64]. In this study, we found 469 AMR determinants in the caeca of aged laying hens, with supplemented layers having a significantly lower proportion of AMR determinants compared with the control. Additionally, there were significant differences in ARG composition between the groups, with 41 of the 49 significantly different genes found to be lower in the supplemented group compared with the control. Previous studies have shown that tetracyclines, macrolides, and aminoglycosides are among the most common antibiotic resistance classes in laying hens, which is consistent with our findings [5,65,66]. 

Most of those genes which were significantly different were determined to be chromosomally located resistance gene determinants, typically efflux pumps, suggesting they are part of the natural resistance gene profile of the core microbiome [67]. Typically, plasmid-mediated resistance genes are thought to pose a greater risk for facilitating the exchange of ARGs under environmental stresses [68]; however, it is argued that chromosomally located ARGs may move into susceptible pathogens and may also pose a risk, making them relevant [69].

Prebiotic mannan-rich fractions modify gut microbial populations and microbial metabolites, inhibiting the colonisation of some bacteria, including type 1 fimbriated pathogens such as *E. coli*, *Shigella*, and *Salmonella*, and other pathogens such as *C. perfringens* and *Listeria* spp., amongst others [8,9,51]. MRF achieves this through competition for binding sites, improving nutrient digestion, intestinal morphology, and function, in addition to regulating the immune response [7,43,52,70]. Pathogenic strains of Enterobacteriaceae and *E. coli* can have an impact on human and animal health by acquiring and disseminating AMR and virulence genes through the food supply chain [71]. Other research has shown that the use of prebiotic mannan-rich fractions has impacted antimicrobial resistance by potentiating bacterial susceptibility to antibiotics in *E. coli* through modulation of the bacterial metabolism [12]. In a previous study which tracked the dynamics of ARG-harbouring bacterial hosts, the authors found that the ARG-harbouring genera in the faecal resistome of broilers belonged to *Escherichia*, *Klebsiella*, *Shigella* and *Salmonella*, with the ARGs resistant to multidrug resistance, tetracycline, aminoglycoside, and MLS being noted to reside in *Escherichia* [66].

A possible explanation for the reduced abundance of ARGs may be through a reduction of these ARG-harbouring bacterial hosts. In our current study, LEfSe analysis showed that Proteobacteria and Enterobacteriaceae were significant discriminators between the control and supplemented groups, with their abundance being over-represented in the control group. In addition, correlation analysis showed that those bacterial genera which were significantly different also had strong and positive correlations between potential pathobionts such as *Escherichia*, *Coprobacter*, *Brachyspira*, and *Trueperella*. Further correlation analysis also showed that an expansion of bacteria that carry fewer AMR genes could be responsible for the observed decrease in the relative abundance of AMR-carrying pathobionts. 

Another study showed that in the chicken metagenome, the proteobacteria carried almost 30% of ARGs, and *Escherichia* was the main ARG host at the genus level [72]. Similar to this, we noted that while the proteobacteria accounted for approximately 4% of the bacteria within the layer caecal content of this study, approximately 12% of resistance genes were associated with this same phylum. According to the CARD database, the phylogenetic associations of the differentially abundant ARGs were mainly Proteobacteria, Enterobacteriaceae, and Bacteroides. In addition, many of these differentially abundant genes were more frequently identified as core genes in the control compared with the supplemented group, where they were identified as part of the accessory resistome. This may be attributed to the shift in microbial composition, where those bacterial hosts typically harbouring these genes were lower and not as frequently identified in the caeca of birds in the supplemented group. These results, taken together with the LefSe and correlation analysis showing proteobacteria and Enterobacteriaceae contribute to the greatest difference between the two bacterial communities, may explain the differences observed in the resistome analysis. 

Interestingly, a large proportion of the ARGs which were differentially abundant in our study (10/49) were associated with tetracycline resistance, with nine of these genes being significantly lower in the supplemented group compared with the control. The same study by Xiong et al. [66] also found that the antibiotic alteration of bacterial community structure significantly influenced ARG-harbouring bacterial hosts via inhibition of *Escherichia* and was the primary reason for an observed decrease in multidrug resistance genes. Tetracycline is one of the most widely used antibiotics in farming. Therefore, there is a need to limit the evolution and spread of tetracycline resistance [73].

## 5. Conclusions

The findings of this commercial field-trial study demonstrate that dietary supplementation with a yeast mannan-rich fraction containing prebiotic product can positively affect laying hen performance, while reducing the observed abundance of antimicrobial resistance genes. We believe the MRF-associated benefits were achieved by a greater abundance of beneficial microbes and simultaneously lowering AMR-carrying pathobionts. Pathogenic strains of Enterobacteriaceae can have an impact on human and animal health by acquiring and disseminating AMR and virulence genes through the food supply chain, leading to increased treatment failures and higher mortality, posing an increasing threat to public health. These changes may limit the evolution and spread of antibiotic resistance genes within the gastrointestinal tract and other environments, such as food and water. 

Future research could explore the underlying mechanisms driving these changes, investigating the specific pathways through which supplementation with a yeast mannan-rich prebiotic influences microbial communities. Additionally, longitudinal studies may provide insights into the temporal dynamics of these effects throughout the laying cycle. Future improvements in monitoring AMR movement will be critical to prevent the spread of resistance genes in the environment, thus contributing to the One Health approach in controlling AMR. Overall, this study greatly contributes to the growing body of knowledge on the beneficial impacts of dietary intervention in poultry and their multifaceted effects on both the microbiome and resistome.

## Figures and Tables

**Table 1:** Impact of supplementation on eggshell strength, egg grading, and performance.

		W24	W30	W38	W45	W52	W59	W63	W70	W75	W24-75
Eggshell strength (kg.f)	Control	5.49	**5.09 a**	**4.92 a**	**4.51 a**	4.43	**4.54 a**	**3.96 a**	4.4	**3.87 a**	**4.55 a**
	Supplemented	5.59	**5.920 b**	**5.24 b**	**5.28 b**	4.88	**5.13 b**	**5.00 b**	4.52	**4.33 b**	**5.10 b**
		W20-29	W30-39	W40-49	W50-59	W60-69	W70-76	W20-76			
ADFI	Control	115.8	127.3	143.6	146.4	125.3	133	130.2			
	Supplemented	117.6	140.2	133.2	129.0	123.5	115.6	127.2			
Mortality	Control	0.019	0.044	0.013	0.089	0.271	0.267	0.063			
	Supplemented	0.019	0.013	0.032	0.057	0.221	0.288	0.051			
Second-grade eggs	Control	4.45	**4.95 a**	**5.95 a**	**8.25 a**	10.27	**10.97 a**	6.51			
	Supplemented	4.36	**4.40 b**	**5.16 b**	**7.48 b**	8.81	**12.39 b**	6.05			

Values represent medians and are compared at each timepoint; values in each column which have different letters (a, b) are significantly different (*p* < 0.05) and are emboldened.

**Table 2:** Differentially abundant antimicrobial resistance genes.

Drug Class	Resistance Mechanism	ARO Name	Control Mean (%)	Supplemented Mean (%)	Log2 Fold Change	Control Genome	Supplemented Genome	Phylogenetic Association
fluoroquinolone antibiotic; cephalosporin; glycylcycline; penam; tetracycline antibiotic; rifamycin antibiotic; phenicol antibiotic; triclosan	antibiotic target alteration; antibiotic efflux	Escherichia_coli_soxR	1.07 × 10^−06^	5.37 × 10^−08^	−4.31	Core	Acc	Proteobacteria
fluoroquinolone antibiotic; cephalosporin; glycylcycline; penam; tetracycline antibiotic; rifamycin antibiotic; phenicol antibiotic; triclosan	antibiotic target alteration; antibiotic efflux	Escherichia_coli_marR	8.34 × 10^−07^	7.18 × 10^−08^	−3.54	Acc	Acc	Bacteria
macrolide antibiotic	antibiotic efflux	mefC	2.19 × 10^−06^	2.16 × 10^−07^	−3.35	Core	Acc	Bacteroidetes; Bacteroides coprophilus CAG:333
fluoroquinolone antibiotic	antibiotic efflux	emrA	8.90 × 10^−07^	8.90 × 10^−08^	−3.32	Acc	Acc	Proteobacteria
aminoglycoside antibiotic	antibiotic efflux	kdpE	8.52 × 10^−07^	8.74 × 10^−08^	−3.28	Acc	Acc	Proteobacteria
aminoglycoside antibiotic; aminocoumarin antibiotic	antibiotic efflux	baeS	7.56 × 10^−07^	8.62 × 10^−08^	−3.13	Core	Acc	Proteobacteria
fluoroquinolone antibiotic; monobactam; carbapenem; cephalosporin; glycylcycline; cephamycin; penam; tetracycline antibiotic; rifamycin antibiotic; phenicol antibiotic; triclosan; penem	antibiotic target alteration; antibiotic efflux; reduced permeability to antibiotic	Escherichia_coli_soxS	1.16 × 10^−06^	1.33 × 10^−07^	−3.12	Acc	Acc	Bacteria
monobactam; carbapenem; cephalosporin; cephamycin; penam	antibiotic target alteration	Haemophilus_influenzae_PBP3	6.65 × 10^−07^	7.67 × 10^−08^	−3.12	Acc	Acc	Proteobacteria
peptide antibiotic	antibiotic efflux	YojI	7.57 × 10^−07^	8.96 × 10^−08^	−3.08	Core	Acc	Proteobacteria
fluoroquinolone antibiotic; cephalosporin; glycylcycline; penam; tetracycline antibiotic; rifamycin antibiotic; phenicol antibiotic; triclosan	antibiotic efflux	acrB	8.20 × 10^−07^	1.02 × 10^−07^	−3.01	Core	Acc	Proteobacteria; Enterobacteriaceae
macrolide antibiotic; fluoroquinolone antibiotic; cephalosporin; cephamycin; penam; tetracycline antibiotic	antibiotic efflux	H-NS	1.02 × 10^−06^	1.27 × 10^−07^	−3.00	Acc	Acc	Proteobacteria
fluoroquinolone antibiotic	antibiotic efflux	mdtH	7.05 × 10^−07^	8.90 × 10^−08^	−2.99	Acc	Acc	Proteobacteria
macrolide antibiotic; fluoroquinolone antibiotic; penam; tetracycline antibiotic	antibiotic efflux	evgS	5.90 × 10^−07^	7.55 × 10^−08^	−2.96	Core	Acc	Proteobacteria; Enterobacteriaceae
phenicol antibiotic	antibiotic efflux	emrD	9.01 × 10^−07^	1.19 × 10^−07^	−2.92	Core	Acc	Proteobacteria
aminocoumarin antibiotic	antibiotic efflux	mdtB	9.06 × 10^−07^	1.21 × 10^−07^	−2.90	Core	Acc	Proteobacteria; Enterobacteriaceae
tetracycline antibiotic; benzalkonium chloride; rhodamine	antibiotic efflux	Escherichia_coli_mdfA	5.75 × 10^−07^	8.33 × 10^−08^	−2.79	Core	Acc	Bacteria
macrolide antibiotic; fluoroquinolone antibiotic; penam	antibiotic efflux	mdtE	9.49 × 10^−07^	1.39 × 10^−07^	−2.77	Acc	Acc	Proteobacteria
tetracycline antibiotic	antibiotic efflux	emrK	5.73 × 10^−07^	9.31 × 10^−08^	−2.62	Acc	Acc	Proteobacteria
fosfomycin	antibiotic target alteration	Escherichia_coli_GlpT	8.14 × 10^−07^	1.36 × 10^−07^	−2.58	Acc	Acc	Proteobacteria
cephalosporin; penam	antibiotic inactivation	Escherichia_coli_ampC	9.61 × 10^−07^	1.61 × 10^−07^	−2.58	Acc	Acc	Proteobacteria/Enterobacteriaceae; Escherichia
peptide antibiotic	antibiotic target alteration	bacA	7.34 × 10^−07^	1.26 × 10^−07^	−2.54	Acc	Acc	Proteobacteria
fosfomycin	antibiotic efflux	mdtG	1.05 × 10^−06^	1.90 × 10^−07^	−2.47	Core	ND	Proteobacteria/Enterobacteriaceae; Escherichia
peptide antibiotic	antibiotic target alteration	PmrF	1.13 × 10^−06^	2.07 × 10^−07^	−2.44	Core	Acc	Proteobacteria
fluoroquinolone antibiotic	antibiotic efflux	patA	9.10 × 10^−07^	1.74 × 10^−07^	−2.39	Core	Acc	Proteobacteria/Firmicutes
nucleoside antibiotic; acridine dye	antibiotic efflux	mdtN	8.21 × 10^−07^	1.59 × 10^−07^	−2.37	Core	Acc	Bacteria
aminocoumarin antibiotic	antibiotic efflux	mdtC	1.32 × 10^−06^	2.84 × 10^−07^	−2.22	Core	Acc	Proteobacteria; Enterobacteriaceae
fluoroquinolone antibiotic; cephalosporin; cephamycin; penam	antibiotic efflux	AcrF	1.63 × 10^−06^	3.82 × 10^−07^	−2.10	Core	Acc	Proteobacteria; Enterobacteriaceae
tetracycline antibiotic	antibiotic efflux	tetL	3.40 × 10^−06^	8.65 × 10^−07^	−1.98	Core	Core	Bacteria
tetracycline antibiotic	antibiotic efflux	tetK	7.68 × 10^−06^	2.25 × 10^−06^	−1.77	Core	Core	Bacteroidetes; Bacteroides
tetracycline antibiotic	antibiotic efflux	tet40	1.00 × 10^−05^	3.71 × 10^−06^	−1.43	Core	Core	Firmicutes; Clostridiales
tetracycline antibiotic	antibiotic target protection	tetBP	1.70 × 10^−06^	6.73 × 10^−07^	−1.34	Core	Core	Bacteria
tetracycline antibiotic	antibiotic target protection	tet44	8.11 × 10^−05^	3.34 × 10^−05^	−1.28	Core	Core	Bacteroidetes; Bacteroides
fluoroquinolone antibiotic	antibiotic efflux	pmrA	1.02 × 10^−05^	4.33 × 10^−06^	−1.24	Core	Core	Bacteroidetes; Bacteroides
tetracycline antibiotic	antibiotic target protection	tetO	1.88 × 10^−05^	8.25 × 10^−06^	−1.19	Core	Core	Bacteria
tetracycline antibiotic	antibiotic target protection	tetW/N/W	2.26 × 10^−04^	1.57 × 10^−04^	−0.52	Core	Core	Bacteria
cephalosporin; penam	antibiotic inactivation	OXA-7	4.71 × 10^−07^	1.03 × 10^−06^	1.13	Acc	Core	Bacteroidetes; Bacteroides
macrolide antibiotic; lincosamide antibiotic	antibiotic target alteration	RlmAII	1.77 × 10^−07^	7.68 × 10^−07^	2.12	Acc	Core	Bacteroidetes; Prevotella bryantii
monobactam; carbapenem; cephalosporin; cephamycin; penam; penem	antibiotic inactivation	GIM-2	3.25 × 10^−07^	0.00 × 10^+00^	Depleted	Acc	ND	Unknown
cephalosporin; penam	antibiotic inactivation	OXA-136	7.55 × 10^−07^	0.00 × 10^+00^	Depleted	Acc	ND	Spirochaetes; Brachyspira pilosicoli
cephalosporin; penam	antibiotic inactivation	OXA-244	1.58 × 10^−07^	0.00 × 10^+00^	Depleted	Acc	ND	Unknown
monobactam; cephalosporin; penam; penem	antibiotic inactivation	TEM-127	1.48 × 10^−07^	0.00 × 10^+00^	Depleted	Acc	ND	Bacteria
monobactam; cephalosporin; penam; penem	antibiotic inactivation	TEM-219	1.39 × 10^−07^	0.00 × 10^+00^	Depleted	Acc	ND	Enterobacteriaceae
streptogramin antibiotic; pleuromutilin antibiotic	antibiotic efflux	vgaC	8.45 × 10^−07^	0.00 × 10^+00^	Depleted	Acc	ND	Proteobacteria; Enterobacteriaceae
aminoglycoside antibiotic	antibiotic inactivation	AAC6-Ii	0.00 × 10^+00^	3.06 × 10^−07^	Detected	Acc	Acc	Unknown
carbapenem; cephalosporin; cephamycin; penam	antibiotic inactivation	ACT-30	0.00 × 10^+00^	4.95 × 10^−07^	Detected	ND	Acc	Unknown
aminoglycoside antibiotic	antibiotic inactivation	APH3-IVa	0.00 × 10^+00^	6.40 × 10^−08^	Detected	ND	Acc	Unknown
penam	antibiotic inactivation	CARB-3	0.00 × 10^+00^	1.55 × 10^−07^	Detected	ND	Acc	Unknown
tetracycline antibiotic	antibiotic efflux	tcr3	0.00 × 10^+00^	1.33 × 10^−07^	Detected	ND	Acc	Ruminococcaceae
streptogramin antibiotic	antibiotic inactivation	vgbC	0.00 × 10^+00^	2.40 × 10^−07^	Detected	ND	Acc	Ruminococcaceae

Genes are shown alongside their mean relative abundance per treatment group, their drug class, resistance mechanism, and phylogenetic associations, and their association with core or accessory (acc) genomes is shown.

## Data Availability

Sequencing data can be found in NCBI Sequence Read Archive (SRA) database (Accession Number: PRJNA1136229). All other data that support the findings of this study are available on request from the corresponding author.

## Supplementary Materials

The following supporting information can be downloaded at: https://www.mdpi.com/article/10.3390/microorganisms12081562/s1, Tables S1–S14.
